# Na,K-ATPase alpha isoforms at the blood-cerebrospinal fluid-trigeminal nerve and blood-retina interfaces in the rat

**DOI:** 10.1186/2045-8118-10-14

**Published:** 2013-03-14

**Authors:** Xianghong Arakaki, Paige McCleary, Matthew Techy, Jiarong Chiang, Linus Kuo, Alfred N Fonteh, Brian Armstrong, Dan Levy, Michael G Harrington

**Affiliations:** 1 Molecular Neurology Program, Huntington Medical Research Institutes, 99 N. El Molino Avenue, Pasadena, CA, 91101, USA; 2 Light Microscopy Core Facility, Beckman Research Institute, City of Hope, 1500 East Duarte Road, Duarte, CA, 91010, USA; 3 Headache Research Laboratory, Department of Anesthesia and Critical Care, Harvard Medical School, 330 Brookline Ave E/CLS 639, Boston, MA, 02115, USA

**Keywords:** Cerebrospinal fluid (CSF), Choroid plexus (CP), Meninges, Retina

## Abstract

**Background:**

Cerebrospinal fluid (CSF) sodium concentration increases during migraine attacks, and both CSF and vitreous humor sodium increase in the rat migraine model. The Na,K-ATPase is a probable source of these sodium fluxes. Since Na,K-ATPase isoforms have different locations and physiological roles, our objective was to establish which alpha isoforms are present at sites where sodium homeostasis is disrupted.

**Methods:**

Specific Na,K-ATPase alpha isoforms were identified in rat tissues by immunohistochemistry at the blood-CSF barrier at the choroid plexus, at the blood-CSF-trigeminal barrier at the meninges, at the blood-retina barrier, and at the blood-aqueous barrier at the ciliary body. Calcitonin gene-related peptide (CGRP), occludin, or von Willibrand factor (vWF) were co-localized with Na,K-ATPase to identify trigeminal nociceptor fibers, tight junctions, and capillary endothelial cells respectively.

**Results:**

The Na,K-ATPase alpha-2 isoform is located on capillaries and intensely at nociceptive trigeminal nerve fibers at the meningeal blood-CSF-trigeminal barrier. Alpha-1 and −3 are lightly expressed on the trigeminal nerve fibers but not at capillaries. Alpha-2 is expressed at the blood-retina barriers and, with alpha-1, at the ciliary body blood aqueous barrier. Intense apical membrane alpha-1 was associated with moderate cytoplasmic alpha-2 expression at the choroid plexus blood-CSF barrier.

**Conclusion:**

Na,K-ATPase alpha isoforms are present at the meningeal, choroid plexus, and retinal barriers. Alpha-2 predominates at the capillary endothelial cells in the meninges and retinal ganglion cell layer.

## Background

Sodium is crucial for neuronal excitability: when the membrane potential of a neuron depolarizes, sodium channels open. When the potential reaches a threshold, the sodium current becomes regenerative and initiates the action potential. Hodgkin and Katz showed that altered extracellular sodium concentration changes the action potential amplitude in squid axon [1,2]. Recent experiments revealed that sodium increases in human lumbar CSF by 3 – 4 mM during migraine attacks [3], and in the intracranial CSF and ocular vitreous humor by over 20 mM in a rat model of central sensitization [4]. Since retinal ganglion cells participate in the photophobia of migraine [5], this intraocular increase of sodium may implicate a local retinal stimulation from elevated sodium concentrations. The degree of these sodium changes are sufficient to increase neuronal excitability by more than 1.75 fold in simulated [4] and primary cultured neurons [6]. Stimulation of trigeminal meningeal nociceptors and retinal neurons might contribute to the cranial pain and light sensitivity of migraine [7]. The enzyme Na,K-ATPase is a potential source to change [Na^+^], and the expression of Na,K-ATPase alpha isoforms in the related barriers will be reported in this study.

Cerebrospinal fluid (CSF) cations are tightly regulated at distinctly different concentrations than in blood, with higher sodium and lower potassium in CSF, made possible because the blood-CSF barrier [8] limits diffusional correction of the gradient. Choroid plexuses (CP) in the lateral, third, and fourth ventricles generate most of the CSF, including these cation gradients. These CP vascular structures have fenestrated capillaries from which the blood exudes to face the basal wall of a single layer of epithelial cells. Tight junctions between these epithelial cells [9] prohibit diffusion from this exudate directly into CSF, and vice versa. The Na,K-ATPase alpha-1 isoform at the apical membrane of these cells [10,11], together with other transporters [12-14] including the Na^+^-K^+^-2Cl^-^ cotransporter and the Na^+^-H^+^ exchanger, is critical for the Na^+^ secretion and K^+^ absorption from CSF that result in this characteristic CSF-blood cation gradient. We, therefore, studied the Na,K-ATPase isoforms at the CP.

Once released from the CP, CSF is in direct contact with capillaries extensively throughout the meninges. Capillaries in the dura, arachnoid, and pia mater have tight junctions [9] in place of their normal fenestrations, although some meningeal vessels have been reported to be fenestrated [15]. Meningeal capillaries, however, have not been as extensively studied as those at the blood–brain capillary interface, where it is clear that surrounding pericytes, astrocyte endfeet, and neuronal processes bolster the endothelial cell tight junctions at the blood–brain barrier [9]. In addition, peripheral sensory nerves have tight junctions between intraneural capillary endothelial cells and the perineurium, forming a blood-nerve-barrier [16], though these have not yet been studied in the meningeal nerves. We define the barrier in meningeal blood vessels between blood and CSF and the barrier in meningeal nerve fibers between nerve and blood/CSF together as the blood-CSF-trigeminal barrier.

The blood supply for the retina includes the intraretinal vessels for the inner 2/3 and choroidal vessels for the outer 1/3. There are two blood-retina barriers: tight junctions between the inner intraretinal capillary endothelial cells at the retinal ganglion cell layer protect the inner 2/3 of the retina, and tight junctions between the retinal pigment epithelial (RPE) cells form the barrier to the outer, fenestrated choroidal blood supply [17-19]. A third barrier in the eye, the blood-aqueous barrier, is formed by the tight junctions in the non-pigmented epithelium (NPE) of the ciliary body [20], where the Na,K-ATPase contributes to sodium and aqueous humor secretion [21].

How sodium dysregulation arises in the migraine model is not defined, and prompted the anatomical studies reported in this paper. Altered Na,K-ATPase activity is considered primarily responsible for the sodium changes found in CSF [7]. The 3 alpha isoforms of Na,K-ATPase in brain have different distributions and activities related to their varied functions, and yet they have not been completely characterized at these blood-fluid barriers. To gain further definition of the Na,K-ATPase isoforms at these barriers, we localize the Na,K-ATPase alpha −1, -2, and −3 isoform expression at the principal source of CSF (in the CP), at the sites of intracranial trigeminal nociception (in the meninges), and at the sites of ocular sodium regulation (in the ciliary body and retina).

## Methods

Fourty-three Sprague–Dawley male rats (Harlan) between the weights of 180 and 240 g were used in these studies. The HMRI Animal Care and Use Committee approved animal procedures.

### Tissue preparation

For the retina, ciliary body, and CP studies, rats were deeply anesthetized with pentobarbital (50–100 mg/Kg). After euthanasia by decapitation, eyes were enucleated and brains were removed. Brain regions were identified with reference to the Paxinos and Watson brain atlas [22]. Coronal sections were cut from eyes and brains, frozen in O.C.T. compound (Tissue Tek, Torrance, CA) in tissue blocks (Pathology Innovations, Wyckoff, NJ), and sliced into 12 μm sections using a cryostat (Mikron, Vista, CA). Each section was mounted on Fisherbrand Superfrost® Plus precleaned slides (Fisher Scientific, Pittsburgh, PA) and stored at −30°C until stained. These tissues were fresh-frozen without cryoprotection, and ethanol fixed on their slide at the start of immunostaining. For meningeal studies, rats were deeply anesthetized with pentobarbital, then transcardially perfused with PBS followed by fresh 2% paraformaldehyde. The dura with meninges were carefully stripped from the inner calvarium, cut around the base of the brain and removed in one piece from its surface. The meninges over the cerebellum were removed; the remainder cut into half, stained as floating sections, and then mounted on slides. This preparation was predominantly dura, but we refer to it as meninges as it also includes significant arachnoid and possibly some pial fragments.

### Antibodies

**Figure 1:**
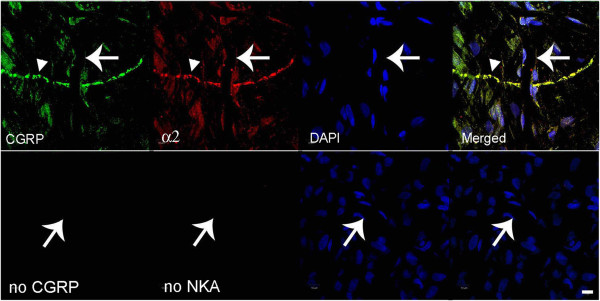
**Fluorescence images of CGRP and Na,K-ATPase alpha-2 in meninges.** In the top row, CGRP is labeled with fluorescein on the left, Na,K-ATPase alpha-2 is visualized with Texas red, nuclei are DAPI-stained, and the merged image is on the right. The large arrow indicates a capillary and the smaller arrowhead indicates a trigeminal nerve. The lower row images are negative controls when no primary antibody was used. The notable finding is that Na,K-ATPase alpha-2 is expressed at the capillary and nerve fiber. Scale bar = 10 μm.

**Figure 2:**
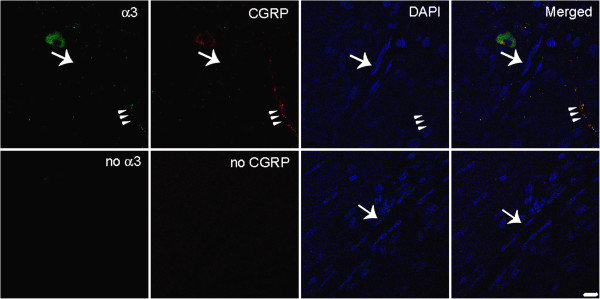
**Fluorescence images of Na,K-ATPase alpha-3 and CGRP in meninges.** In the top row, Na,K-ATPase alpha-3 is labeled with fluorescein in the left, CGRP is visualized with Texas red, nuclei are DAPI-stained, and the merged image is on the right. The large arrow indicates a capillary and the arrowhead triplet indicate a trigeminal nerve. The lower horizontal images are negative controls when no primary antibody was used. The notable finding is that Na,K-ATPase alpha-3 is expressed at the nerve fiber, not on the capillary. Scale bar = 10 μm.

**Figure 3:**
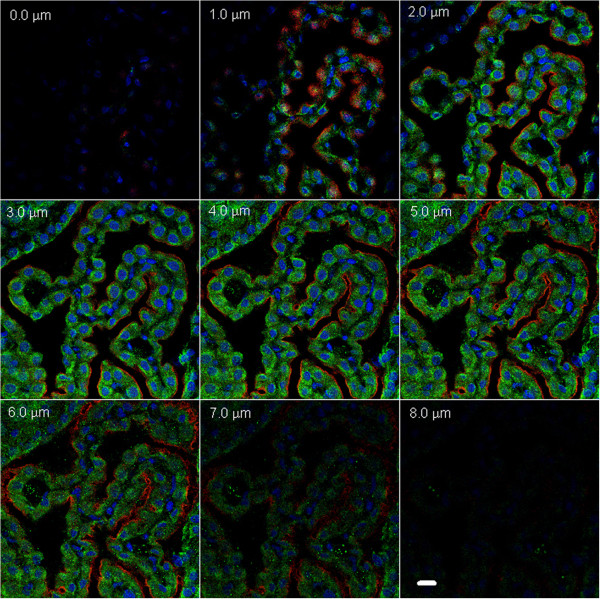
**Representative immunofluorescence images of a choroid plexus from the 3rd ventricle, sequentially acquired at 1 μm steps in a z-stack to reveal the cellular location of Na,K-ATPase alpha-1 and −2 isoforms.** The same Na,K-ATPase expression patterns were found in the choroid plexuses in the lateral ventricles (data not shown). Texas red is used to visualize alpha-1 and fluorescein for alpha-2, and the nuclei are DAPI stained. Negative controls (without primary antibody; data not shown) had no membrane or cytoplasmic staining. Scale bar = 10 μm.

**Figure 4:**
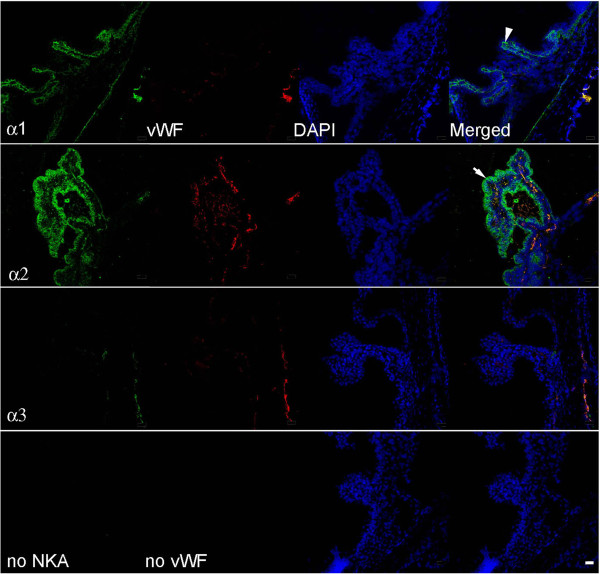
**Representative immunofluorescence images of the blood-aqueous barrier at the rat ciliary body with Na,K-ATPase alpha-1, -2, and −3 isoform expression.** Na,K-ATPase isoforms labeled with fluorescein in left hand column images, vWF visualized with Texas red in second column, nuclei are DAPI stained, and the merged image for each horizontal panel is in the right hand column. Lower horizontal images are negative controls with no primary antibody. Merged images show alpha-1 expressed on the PE, alpha-2 on the NPE, and alpha-2 expressed on ciliary body capillaries. The arrowhead points to the PE on the alpha-1 merged image, and the arrow points to the NPE on the alpha-2 merged image. Scale bar = 20 μm.

**Figure 5:**
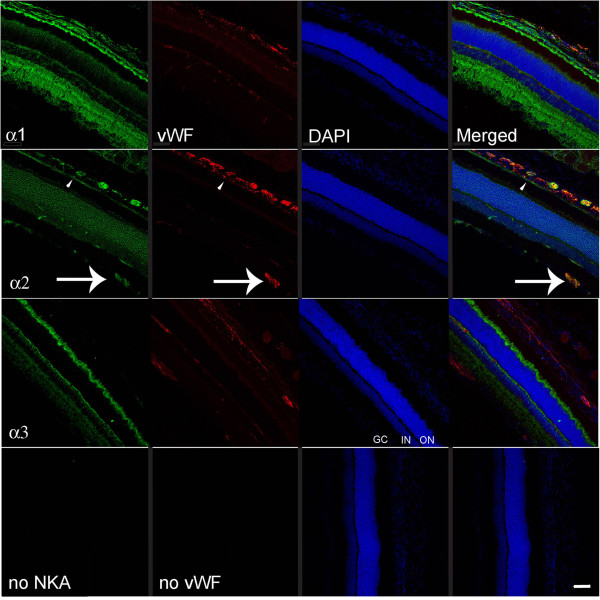
**Representative immunofluorescence images of Na,K-ATPase alpha-1, -2, and −3 expression at the rat retina.** Na,K-ATPase isoforms labeled with fluorescein in the left hand column images, vWF visualized with Texas red in the second column, nuclei are DAPI-labeled, and the merged image for each horizontal panel is in the right hand column. Lower horizontal images are negative controls when no primary antibody was used. Unique Na,K-ATPase alpha-1 & -3 specific expression in the retinal cell layers is seen, similar to previous reports [23,44]. The arrow indicates alpha-2 expression in the capillaries at the retinal ganglion cell layer. GC: ganglion cell layer; IN: inner nuclear layer; ON: outer nuclear layer are labeled for orientation on the alpha-3 DAPI image. Scale bar = 50 μm.

The following antibodies were used in this study: mouse monoclonal anti-Na,K-ATPase alpha-1 (Cat# 05–369, Millipore); rabbit anti-Na,K-ATPase alpha-2 (Cat# 07–674, Millipore); rabbit anti-Na,K-ATPase alpha-3 (Cat#: 06–172, Millipore); mouse monoclonal anti-vWF (Cat# LS-B4034, Lifespan Biosciences); rabbit anti-vWF (Cat#: ECM 590, Millipore); guinea pig anti-CGRP (Cat#: T-5027, Peninsula Laboratories, LLC); rabbit anti-occludin (Cat#: sc-5562, Santa Cruz Biotechnology); goat anti-guinea pig IgG (Cat#: BA-7000, Vector Laboratories); goat anti-rabbit IgG (Cat# BA-1000; Vector Laboratories); horse anti-mouse rat adsorbed IgG (Cat# BA-2001, Vector Laboratories). The following combinations were used in fluorescence experiments: For Figure 1, guinea pig anti-CGRP primary antibody, goat anti-guinea pig IgG secondary antibody, with fluorescein; followed by rabbit anti-Na,K-ATPase alpha-2 primary antibody (1:500), goat anti-rabbit IgG secondary antibody, with Texas red. For Figure 2, guinea pig anti-CGRP primary antibody, goat anti-guinea pig IgG secondary antibody, with Texas red; followed by mouse anti-Na,K-ATPase alpha-1 primary antibody, horse anti-mouse rat adsorbed IgG secondary antibody, with fluorescein. For Figure 3, rabbit anti-Na,K-ATPase alpha-2 primary antibody, goat anti-rabbit IgG secondary antibody, with fluorescein; followed by mouse anti-Na,K-ATPase alpha-1 primary antibody, horse anti-mouse rat adsorbed IgG secondary antibody, with Texas red. For Figures 4 and 5, mouse anti-Na,K-ATPase alpha-1, or rabbit anti-Na,K-ATPase alpha-2, or rabbit anti-Na,K-ATPase alpha-3 primary antibody, horse anti-mouse rat adsorbed IgG (for alpha-1) or goat anti-rabbit IgG (for alpha-2 and alpha-3) secondary antibody, with fluorescein; followed by rabbit anti-vWF (for alpha-1) or mouse anti-vWF (for alpha-2 and alpha-3) primary antibody, goat anti-rabbit IgG (for alpha-1) or horse anti-mouse (for alpha-2 and alpha-3) IgG secondary antibody, with Texas red. To reduce background, all wash solutions contained 2% normal serum from the host of the secondary antibody. Since the dura is around 50 μm thick and tends to have high background, all dura rinses between treatments were 4 times15 min.

The aforementioned Na,K-ATPase alpha-2 antibody we used was imperfect for retinal layer staining in comparison to the published work [23], but we demonstrate its specificity on retinal capillaries in the retinal ganglion cell layer and CP.

### Immunohistochemistry

Meninges, CPs from the lateral and 3rd ventricles, and eyes were labeled with antibodies specific for the Na,K-ATPase alpha-1, alpha-2, and alpha-3 isoforms (1:1,000 unless otherwise stated, Millipore, Billerica, MA), von Willebrand factor (vWF) (1:500 dilution from Millipore or 1:100 dilution from Lifespan Biosciences, Seattle, WA), calcitonin gene-related peptide (CGRP) (1:5000 in Figure 2, 1:1,000 dilution in all other occasions, Peninsula Laboratories, LLC, San Carlos, CA), and occludin (1:200 dilution from Santa Cruz Biotechnology, Inc., Santa Cruz, CA). Species-specific, biotinylated secondary antibodies were optimized at 1:200 or 1:500. Negative control procedures included identical steps, but without primary antibody. Pre-absorption of the alpha-2-antiserum with peptide in molar excess abrogated specific immunoreactive alpha-2 at the CP epithelium, the retinal ganglion cell layer, and the retinal pigment epithelium.

*Diaminobenzidine tetrahydrochloride (DAB) staining*: after washing out the fixative with phosphate-buffered saline containing 0.5% Triton X-100 (PBST), meninges were incubated in avidin for 15 min, rinsed with PBST, biotin blocking solution (Vector Laboratories, Burlingame, CA) for 15 min, followed by 1–2 h in a solution of 5% normal serum (from same species as the host of the secondary antibody) and 0.3% H_2_O_2_ in PBST. Slices were incubated in primary antibody (alpha-1, alpha-2, alpha-3, CGRP, occludin, and vWF) or peptide pre-absorbed alpha-2 antibody with 2% normal serum in PBST at 4°C overnight, washed in PBS, incubated for 2 h at room temperature with biotinylated secondary antibody with 2% normal goat serum in PBS, washed in PBS, incubated in freshly prepared ABC complex (Vector laboratories) for 30 min, washed in PBS, incubated in DAB (Invitrogen Life Technologies, Grand Island, NY) solution for 5–10 min, and rinsed in PBS to terminate the reaction. The edges of meninges were scored to flatten their curve and mounted on glass slides. After drying overnight, meninges were dehydrated with ethanol (70, 95, 100, 100%; 10 min each step), xylene for 10 min, and covered in mounting medium (Thermo Scientific) with cover slides.

*Fluorescence double labeling*: Eyes and brain slides were fixed in 100% ethanol for 2 h. Fixed tissues on slides or freshly prepared, floating meninges were stained using the aforementioned procedure but replacing the ABC complex with 30 min to 1 h incubation in Fluorescein Avidin DCS, or Texas Red Avidin D (Vector Laboratories). This and all subsequent procedures were carried out in the dark. After PBST washes, the same process was repeated for the second antibody and the opposite fluorophore from that used for the first antibody. The slices were rinsed in PBS, mounted with ProLong Gold antifade reagent with 4’, 6-diamidino-2-phenylindole (DAPI) (Invitrogen, Sunnyvale, CA), a cover slide was attached and sealed with clear fingernail polish. Preparations were cured in darkness, usually for > 24 h.

**Figure 6:**
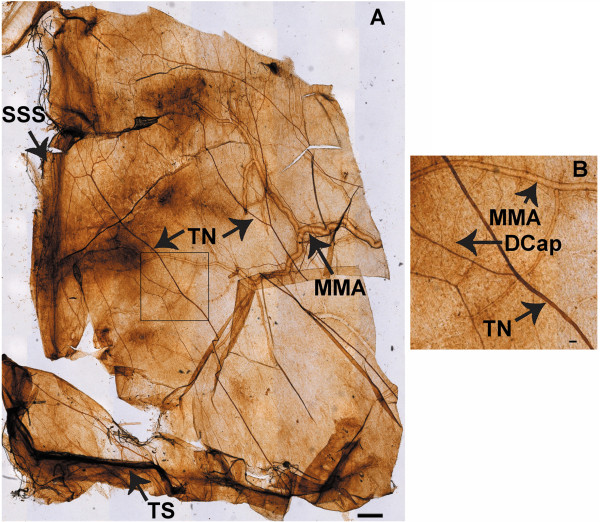
**A is a tiled DAB image of Na,K-ATPase alpha-2 immunostaining at the rat meninges.** The field of view is half of the meninges, extending from the transverse sinus (TS) to the superior sagittal sinus (SSS) centrally and the middle meningeal artery (MMA) laterally, with the trigeminal nerve fibers (TN) crossing the dura. Scale bar 500 μm. **B**: Enlarged region of meninges, magnified from the rectangle area in Figure 9A, illustrating branches of the MMA, a TN, and a capillary (DCap). Scale bar = 50 μm. For control, see Figure 10.

**Figure 7:**
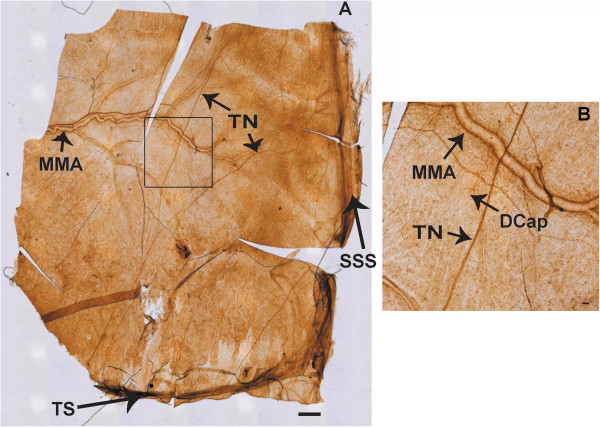
**A is a tiled DAB image, locating occludin at the rat meninges.** The field of view is half of the meninges, extending from the TS to the SSS centrally, the MMA laterally, with the TN crossing the dura. Scale bar 500 μm. **B**: Enlarged region of meninges, magnified from the rectangle area in Figure 6A, illustrating branches of the MMA, a TN, and a capillary (DCap). Scale bar = 50 μm. For control, see Figure 10.

**Figure 8:**
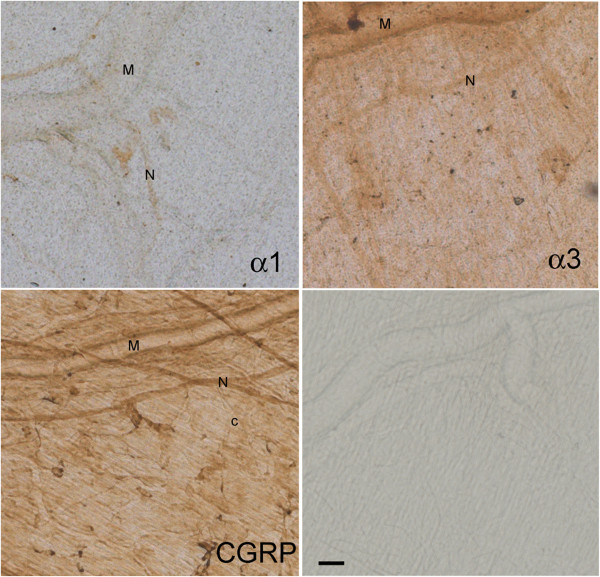
**Representative images of meningeal areas showing DAB-stained Na,K-ATPase alpha-1, alpha-3, CGRP, and control, as indicated in the sections.** M: branch of middle meningeal artery; N: trigeminal nerve fiber; c: capillary. Scale bar = 50 μm.

DAB-stained sections were imaged on a Nikon eclipse TS100 microscope. Large tiled images (up to 8x8 tiles, See Figures 1 &6) were acquired on a Zeiss Observer Z1 microscope (Carl Zeiss, Jena, Germany), 5x/0.5NA objective, Zeiss AxioCamMrC5 color camera, and AxioVision software v.4.8. Fluorescence images were captured on a Zeiss LSM 510 NLO microscope (Carl Zeiss, Jena, Germany) using a 20x/0.8 or 63x/1.4 lens. Samples were illuminated with Argon/ion (488nm), HeNe (543nm), and Chameleon Ultra 2-photon (790nm) lasers. Subtraction of paired images (Figures 7 &8) was achieved using MIPAV (http://mipav.cit.nih.gov, Version 12.5).

## Results

### Meninges

**Figure 9:**
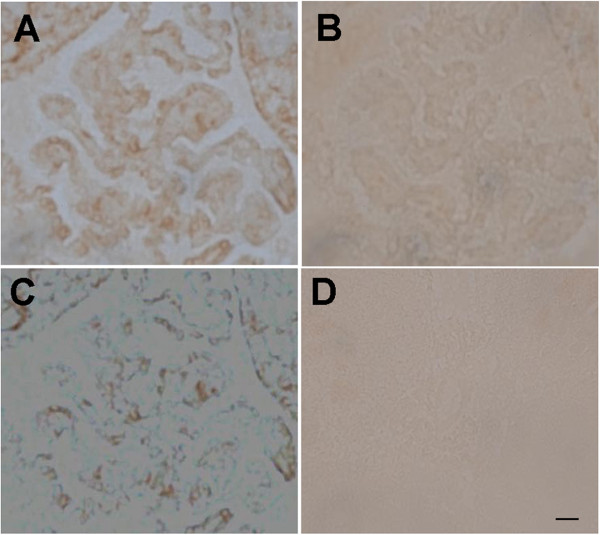
**Specificity of Na,K-ATPase alpha-2 immunoreactivity at the choroid plexus.** The CP was labeled with anti-alpha-2 (**A**) or with peptide pre-absorbed anti-alpha-2 (**B**). The subtraction image is in **C**. **D**: no primary control. Scale bar = 20 μm.

**Figure 10:**
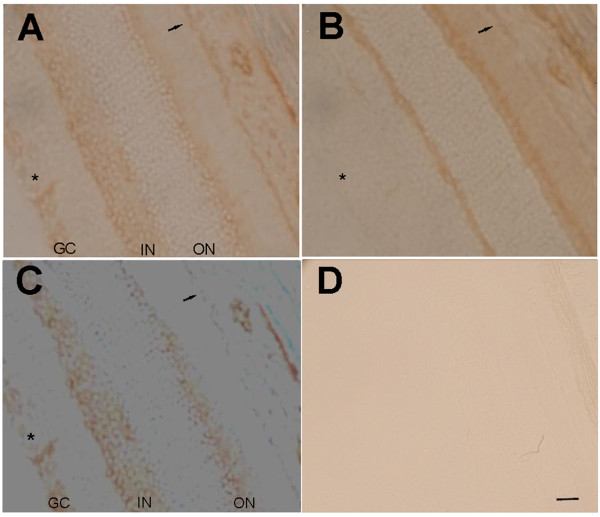
**Specificity of Na,K-ATPase alpha-2 immunoreactivity at the retina.** The retina was stained with anti-alpha-2 (**A**) or with pre-absorbed anti-alpha-2 (**B**). Subtraction image is shown in **C**. **D**: no primary negative control. GC: ganglion cell layer; IN: inner nuclear layer; ON: outer nuclear layer; asterisk: ganglion cell layer; small arrow: RPE. Scale bar = 20 μm.

We used CGRP with immunoreactivity for trigeminal meningeal nerve fibers, some of which are on or near meningeal vessels [24], and because they are altered in migraine [25]. We observed that Na,K-ATPase alpha-2 was expressed in trigeminal nerve fibers, the transverse and sagittal sinuses, large middle meningeal artery branches (Figure 9), and capillaries (Figure 9B, Figure 1). The tight junction protein, occludin, was also expressed on trigeminal meningeal nerve fibers, venous sinuses, MMA branches, and capillaries (Figure 6), with the same distribution as Na,K-ATPase alpha-2 (Figure 9) and CGRP (Figure 10). The anti-CGRP stained the nerve fiber, but not the capillaries in Figure 2, probably because the concentration is much lower (1:5,000), and Texas red, a weaker signal than fluorescein, was used for CGRP in this experiment. Na,K-ATPase alpha-1 and alpha-3 were lightly expressed in MMA branches and nerve fibers, but not at capillaries (Figure 10). Alpha-3 expression was confirmed in fluorescence labeling at trigeminal nerve fibers, but not at capillaries (Figure 2).

### Choroid plexus

Na,K-ATPase alpha-1 was intensely expressed along the apical membrane of the choroidal epithelial cells, while the alpha-2 was moderately expressed in epithelial cell cytoplasm. The cellular locations of these isoforms are distinct on the z-stacked images (Figure 3). Na,K-ATPase alpha-2 labeling in the CP was abrogated with peptide pre-absorption (Figure 7), confirming the specificity of the cytoplasmic alpha-2 immunoreactivity. Na,K-ATPase alpha-2 was also expressed in ventricular ependymal cells and in the capillaries of the CP (Figure 7 and data not shown). Na,K-ATPase alpha-3 was not expressed in the choroid epithelium or in its blood supply (data not shown).

### Ciliary body

The Na,K-ATPase alpha-1 isoform is expressed on the basolateral surface of the pigmented epithelium (PE), while alpha-2 is located densely in the basolateral membrane of the NPE on the side facing the aqueous humor. Furthermore, the alpha-2 co-localized with vWF, the endothelial cell marker, in the ciliary body capillaries. Alpha-3 was not expressed in the ciliary body epithelium or in the capillaries (Figure 4).

### Retina

Na,K-ATPase alpha-1 and alpha-2 were expressed in the RPE, but only the alpha-2 isoform co-localized with vWF on the blood vessel endothelial cells around the retinal ganglion cells (Figure 5). The alpha-3 isoform did not co-localize with any blood vessels in the retina. After peptide pre-absorption, alpha-2 expression in the ganglion cell layer and RPE disappeared, indicating specificity of alpha-2 immunoreactivity at these locations (Figure 8).

## Discussion

We report that the ouabain-sensitive Na,K-ATPase alpha-2 isoform is located at CP, meningeal, retinal, and ciliary body barriers, either by itself or with alpha-1, and the alpha-2 is the consistent capillary endothelial cell isoform at these blood-barrier locations. The endothelial cell does not always choose the Na,K-ATPase alpha-2 in the nervous system; for instance, the alpha-1 is the endothelial isoform in the labyrinthine barrier [26]. It is plausible that the specific Na,K-ATPase isoforms at these locations are important in sodium flux, and thus contribute to the sodium disturbance of migraine. One distinction for Na,K-ATPase regulation conferred by alpha chain isoforms is that alpha-1 is relatively insensitive to ouabain (K_d_ around 30 μM), whereas alpha-2 and −3 are sensitive with K_d_ around 15 – 41 nM [27], close to the concentration of circulating endogenous ouabain-like compounds [28]. In humans, the Na,K-ATPase K_d_ for ouabain appears similar for the three alpha isoforms in studies thus far, while varying considerably between different cardiac glycoside compounds [29].

### Meninges

The meningeal barrier is created at the tight junctions between the meningeal vascular endothelial cells, separating capillary blood from CSF, and at the trigeminal nerve barrier. The faint presence of Na,K-ATPase alpha-1 and −3 isoforms in meningeal sensory nerve fibers and the intense alpha-2 isoform expression in both meningeal nerve fibers and capillaries indicate an important neurovascular role for Na,K-ATPase alpha-2 in regulating sodium at the trigeminal nerves. These results provide the first evidence that both occludin and alpha-2 Na,K-ATPase are expressed in the rat meninges, both in blood vessels and around CGRP-positive, nociceptive nerve fibers. Occludin expression in the nerve fiber is most likely in the endoneurial capillary endothelial cells and perineurium as previously reported in peripheral nerves [16]. Precise localization of Na,K-ATPase alpha-2 and occludin within the meningeal nerve fiber compartments will require further study.

Our studies offer anatomical support for Na,K-ATPase alpha-2 as a modulator of intra-meningeal sodium at the blood-CSF-trigeminal barrier and, to a lesser extent, for alpha-1 and −3 at the trigeminal nerve fibers.

### Choroid plexus

Consistent with previous studies in the CP [10,11], we found the alpha-1 subunit is intensely expressed in the apical membrane of the epithelial cells. The alpha-1 isoform has substantially lower affinity (K_d_ in the μM range) for the specific Na,K-ATPase inhibitor, ouabain, than is required to inhibit the alpha-2 and −3 Na,K-ATPase (K_d_ in the nM range), consistent with the demonstration that 1 mM ouabain is required to retain sodium in the CP [30]. Using both immunocytochemistry and *in situ* hybridization, CP apical membrane Na,K-ATPase alpha1 and AQP1 were reported to decrease with age, thus decreasing CSF secretion in aged rats [11]. Zlokovic and colleagues reported alpha-1, beta-1, and beta-2 in the rat CP, but not alpha-2 [31]. Multiple lines of evidence, therefore, indicate that the apical epithelium Na,K-ATPase alpha-1 is critical for CSF and sodium regulation at the choroidal blood-CSF barrier.

The CP barrier, however, may have more complex regulation of sodium homeostasis, as we found moderate cytoplasmic Na,K-ATPase alpha-2 expression at choroidal epithelial cells. Since our CP alpha-2 immunoreactivity is not consistent with the previous study [31], we pre-absorbed alpha-2 binding sites with excess peptide, and the results confirm cytoplasmic alpha-2 expression (Figure 7). Furthermore, we defined the cellular localization of the two alpha isoforms with 2-photon microscopy (Figure 3). The difference between our results and previous reports might arise from different methods: tissue preparation — previous authors used fixed tissues, while we stained fresh slices of retina; antibodies — previous authors used polyclonal antibodies derived from purified rat brainstem Na,K-ATPase preparations (no longer available), while we used synthetic peptide-derived commercial antibodies that are not as specific immunohistochemically for retinal neurons, but are specific for capillaries; sensitivity — some authors used Western blotting while we used fluorescence immunostaining.

CSF production and Na,K-ATPase in the CP are regulated at many levels. Studies have demonstrated that 5-HT or noradrenaline, known migraine triggers, can reduce CSF production, an activity that was synergistically inhibited by 5-HT and isoproterenol through PKC, or inhibited through PKA [32]. Cholinergic agents via the NO/cGMP pathway have been shown to inhibit CP Na,K-ATPase activity in bovine studies [33]. Thus, modulation of Na,K-ATPase activity is important for CP functions, and includes pathways known to be involved in migraine.

The overall regulation by alpha, beta, or gamma subunits of the CP Na,K-ATPase at the blood-CSF barrier remains to be elucidated, but we concur with previous studies that the Na,K-ATPase alpha-1 is the primary regulator of CSF sodium at the CP epithelium, while the role for alpha-2 in the CP epithelial cytoplasm merits further study.

### Ciliary body

Na,K-ATPase in the ciliary body is modulated by various factors that affect intraocular pressure and aqueous fluid [34-36]. Our finding that Na,K-ATPase alpha-2 is expressed in the endothelial cells of the fenestrated capillaries, and very densely in the NPE, while alpha-2 is expressed in the PE, is consistent with previous work [21]. Earlier work suggested that the Na,K-ATPase alpha-1 in PE might control overall sodium secretion, and alpha-2 in the NPE may be more responsive to environmental factors [37].

### Retina

Our vWF expression data is consistent with the previously described distribution of intraretinal blood vessels [38]. The Na,K-ATPase alpha-1, -3 expression we find in the retina is also consistent with previous reports [23]. The Na,K-ATPase alpha-2 expression (Figure 8), however, does not match the immunoreactivity shown in the retinal layers in these earlier studies (with a different antibody). Nevertheless, peptide pre-absorption demonstrates alpha-2 specific expression strongly in the ganglion cell layer and modestly at the RPE. The Na,K-ATPase expression in the rat RPE is also consistent with previous light and electron microscopy, and cell culture studies [39,40], however, we are cautious about interpreting our modest RPE specific immunoreactivity: previous immunoblotting demonstrated alpha-1, beta-1, and beta-2 Na,K-ATPase subunits from human RPE cells [41], but these authors found no alpha-2 or −3 RNA transcripts in the human.

Dysregulation of retinal Na,K-ATPase leads to many pathophysiologies [42,43]. Our findings suggest that the Na,K-ATPase alpha-2 isoform in the blood-retinal barrier at the retinal ganglion cell layer may play a critical role in maintaining sodium homeostasis in the retina.

## Conclusions

1. Our study provides anatomical evidence of Na,K-ATPase, mainly the alpha-2 isoform, at the meningeal trigeminal nerve fibers and capillaries, and at the retinal ganglion cell layer.

2. At the CP blood-CSF and ciliary body blood-aqueous barriers, the alpha-1 is the dominant Na,K-ATPase, though alpha-2 is also present.

## Abbreviations

CGRP: Calcitonin gene-related peptide; CSF: Cerebrospinal fluid; CP: Choroid plexus; DAB: Diaminobenzidine tetrahydrochloride; NPE: Non-pigmented epithelium; Oatp2: Organic anion transporting polypeptide 2; PE: Pigmented epithelium; RPE: Retinal pigment epithelium; vWF: Von willebrand factor

## Competing interests

The authors declare that they have no competing interests.

## Author contributions

XA was involved in study design, data acquisition, data analysis, and helped to draft and critically review the manuscript. PM was involved in data acquisition and data analysis. MT, JC, and LK participated in data acquisition. AF participated in study design and critically reviewed the manuscript. BA participated in data acquisition. DL helped in dura preparation and immunostaining, and critically reviewed the manuscript. MH designed the study, was involved in data acquisition, data analysis, and wrote the manuscript. All authors helped review the manuscript, and approved the final version of the manuscript.
